# Rotavirus capping enzyme VP3 inhibits interferon expression by inducing MAVS degradation during viral replication

**DOI:** 10.1128/mbio.02255-23

**Published:** 2023-10-31

**Authors:** Jin Dai, Chantal A. Agbemabiese, Ashley N. Griffin, John T. Patton

**Affiliations:** 1 Department of Biology, Indiana University, Bloomington, Indiana, USA; Virginia Polytechnic Institute and State University, Blacksburg, Virginia, USA

**Keywords:** rotavirus, viral protein 3, nonstructural protein 1, interferon, mitochondrial antiviral signaling protein, interferon regulatory factor 3

## Abstract

The binding of viral RNA to RIG-I-like receptors triggers the formation of mitochondrial antiviral signaling (MAVS) protein aggregates critical for interferon (IFN) expression. Several rotavirus strains have been shown to suppress IFN expression by inducing MAVS degradation. Relying on transient expression assays, previous studies reached different conclusions regarding the identity of the rotavirus protein responsible for MAVS degradation, suggesting it was an activity of the rotavirus capping enzyme VP3 or the interferon antagonist NSP1. Here, we have used recombinant SA11 rotaviruses to identify the endogenous viral protein responsible for MAVS degradation and to analyze how the attack on MAVS impacts IFN expression. The recombinant viruses included those expressing modified VP3 or NSP1 proteins deficient in the ability to induce the degradation of MAVS or interferon regulatory factor-3 (IRF3), or both. With these viruses, we determined that VP3 directs the proteasomal degradation of MAVS but plays no role in IRF3 degradation. Moreover, NSP1 was determined to induce IRF3 degradation but to have no impact on MAVS degradation. Analysis of rotavirus-infected cells indicated that IRF3 degradation was more efficient than MAVS degradation and that NSP1 was primarily responsible for suppressing IFN expression in infected cells. However, VP3-mediated MAVS degradation contributed to IFN suppression in cells that failed to produce functional NSP1, pointing to a subsidiary role for VP3 in the IFN antagonist activity of NSP1. Thus, VP3 is a multifunctional protein with several activities that counter anti-rotavirus innate immune responses, including capping of viral (+)RNAs, hydrolysis of the RNase L 2–5A (2′−5′ oligoadenylate) signaling molecule, and proteasomal degradation of MAVS.

**IMPORTANCE:**

Rotavirus is an enteric RNA virus that causes severe dehydrating gastroenteritis in infants and young children through infection of enterocytes in the small intestine. Timely clearance of the virus demands a robust innate immune response by cells associated with the small intestine, including the expression of interferon (IFN). Previous studies have shown that some rotavirus strains suppress the production of interferon, by inducing the degradation of mitochondrial antiviral signaling (MAVS) protein and interferon regulatory factor-3 (IRF3). In this study, we have used reverse genetics to generate recombinant rotaviruses expressing compromised forms of VP3 or NSP1, or both, to explore the function of these viral proteins in the degradation of MAVS and IRF3. Our results demonstrate that VP3 is responsible for MAVS depletion in rotavirus-infected cells, and through this activity, helps to suppress IFN production. Thus, VP3 functions to support the activity of rotavirus NSP1, the major interferon antagonist of the virus.

## INTRODUCTION

Mucosal surfaces serve as major entry portals for viruses into the human host (1, 2). The key to controlling such infections is the expression of interferon (IFN) by mucosal epithelial cells and associated immune cells (3). Infection of mucosal cells by RNA viruses is often sensed by either of two cytoplasmic retinoic acid-inducible gene I (RIG-I)-like receptors, RIG-I or melanoma differentiation-associated protein 5 (MDA-5), through their ability to recognize structural features typical of viral RNAs (4–6). The activation of these RNA sensors triggers the activation of the mitochondrial antiviral signaling (MAVS) protein, which as a result undergoes extensive oligomerization on the mitochondrial membrane, forming filamentous aggregates (7). The recruitment of kinases and other factors to MAVS aggregates generates multifunctional signalosome complexes with activities that include inducing the phosphorylation and dimerization of IFN-regulator factor-3 (IRF3) (8). The activated form of IRF3 undergoes nuclear translocation where it triggers the expression and secretion of IFNs (9). Through autocrine and paracrine signaling, the IFNs upregulate the expression of IFN-stimulated genes (ISGs) products with diverse antiviral activities capable of inhibiting virus replication (10). Viruses have evolved a wide variety of mechanisms to subvert the expression of IFNs and ISGs, which includes preventing the function of MAVS. For example, the HBX protein of hepatitis B virus promotes K136-linked ubiquitination and proteasomal degradation of MAVS (11); the ORF-9b of coronavirus SARS-CoV catalyzes K48-linked ubiquitination and proteasomal degradation of MAVS and other components of MAVS/TRAF3/TRAF6 signalosome (12); the serine protease, NS3/4A, produced by hepatitis C virus cleaves MAVS, dislocating it from the mitochondrial membrane (13–15); and the cysteine protease 2A^pro^ encoded by enterovirus EV71 cleaves MAVS at multiple sites, causing its loss from the mitochondrial membrane (16).

Rotaviruses are segmented double-stranded RNA viruses that belong to the *Sedoreoviridae* family (17). These viruses are the leading cause of severe dehydrating gastroenteritis in infants and young children around the world. The primary target of rotavirus infection is the mature enterocytes located at the villus tips of the small intestine (18). Like most RNA viruses, rotavirus is sensitive to the antiviral effects brought about by IFN and ISG expression (19, 20). The rotavirus proteins, NSP1 and VP3, work to counter IFN and ISG expression by inducing the degradation of transcription factors and other host components necessary for effective innate immune responses (21, 22). For example, many animal strains of rotavirus, such as simian SA11, are known to encode NSP1 proteins that induce the degradation of IRF3. By contrast, the NSP1 proteins of many human rotavirus strains, such as Wa, target β−TrCP (β−transducin repeat-containing protein) for degradation, a host protein necessary for the activation of NF-κB (nuclear factor kappa-light-chain-enhancer of activated B cells) (23). In addition, some forms of NSP1 can block the activity of the antiviral 2′−5′-oligonucleotide synthetase (OAS)-RNase L pathway by inducing the degradation of RNase L (24). Rotavirus VP3, the viral RNA capping enzyme (25), also acts to inhibit the OAS-RNase L pathway, using its C-terminal 2′−5′ phosphodiesterase (PDE) domain which degrades the OAS signaling molecule, 2′−5′ oligoadenylate (2–5A) (26, 27).

Inhibition of MAVS expression in rotavirus-infected cells and mice has been correlated with decreased IFN production and enhanced virus replication (6, 28). Two previous reports have indicated that some rotavirus strains can counter IFN production by inducing the degradation of MAVS (29, 30). Both reports relied on transient expression assays as the principal means of identifying viral proteins affecting levels of MAVS. One report concluded that rotavirus NSP1, by redirecting the targeting activity of host E3 ubiquitin ligases, can induce the ubiquitination and proteasomal degradation of MAVS (29). This suggestion parallels studies showing that NSP1 can direct the proteasomal degradation of other proteins connected to the expression of IFN and ISGs, including IRF3, β−TrCP, and RNase L (24, 31, 32). Another report concluded that, instead of NSP1, VP3 was responsible for rotavirus-induced MAVS degradation (30, 32). Evidence was provided through deletion mutagenesis that the N-terminal region of VP3 was responsible for directing an interaction with MAVS that leads to MAVS degradation (30). Other activities of VP3 have been linked to suppress the expression and activities of IFN and ISGs. Notably, VP3 is the rotavirus RNA capping enzyme (25) and adding a cap-1 structure to viral plus-sense (+)RNAs, including a 2′O methyl group, helps the virus avoid sensing by RIG-I (33, 34). VP3 also contains the PDE domain that antagonizes the OAS-RNase L pathway (35).

In this study, we have used recombinant rotaviruses to identify the viral protein responsible for MAVS degradation in infected cells and to analyze how the attack of MAVS impacts IFN expression. Key to this study was the generation of recombinant viruses that failed to induce the degradation of MAVS or IRF3, or both. Our results demonstrate that VP3 is responsible for MAVS depletion in the infected cell, and through this activity, helps to suppress IFN production. Thus, VP3 functions as a supplement to the activity of rotavirus NSP1, the major interferon antagonist of the virus.

## RESULTS

### Correlation of rotavirus VP3 with MAVS degradation

**Fig 1:**
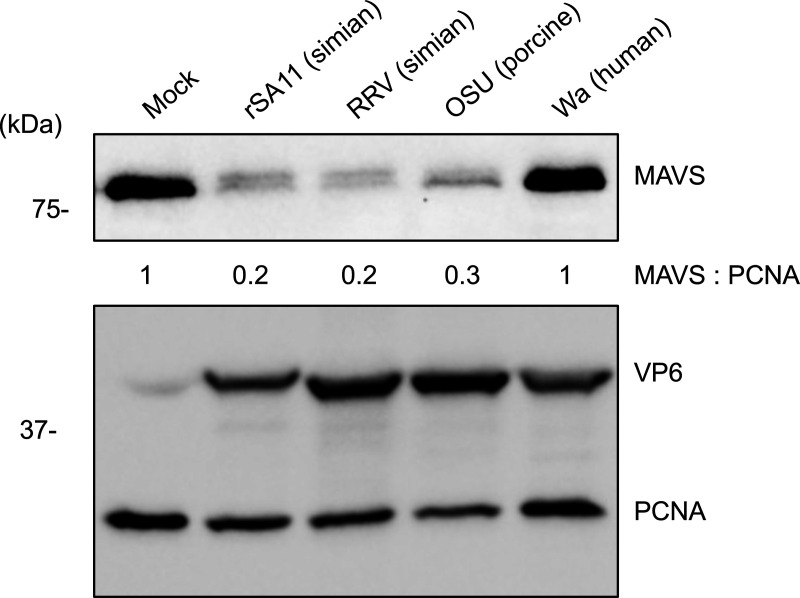
Degradation of MAVS in rotavirus-infected cells. HT-29 cells were mock infected or infected with rotavirus strains at an MOI of 5. Immunoblot assay was used to detect MAVS, VP6, and PCNA in cell lysates prepared at 8 h p.i. Band intensities were determined with Image J software and used to calculate the ratio of MAVS:PCNA. The ratio of MAVS band intensity relative to PCNA for mock-infected cells was set to 1. Results are representative of three independent experiments. Prior to infection, viruses were activated by incubation with 2.5 µg/mL trypsin for 1 hour at 37°C.

To gain greater insight into the loss of MAVS in rotavirus-infected cells, we infected cells and examined the ability of four commonly used rotavirus strains (simian rSA11, simian RRV, porcine OSU, and human Wa) to affect MAVS protein levels in human colorectal epithelial HT-29 cells. HT-29 cells infected with each of the four strains at a multiplicity of infection (MOI) of 5 were harvested at 8 hours post-infection (h p.i.). The infected cell lysates were analyzed by immunoblot analysis using an anti-MAVS antibody. Mammalian cells contain two forms of MAVS, a full-length MAVS (~75 kDa) and an N-terminal truncated mini-MAVS (~52 kDa), both of which are produced from the same bicistronic transcript (36). Full-length MAVS is essential for IFN signaling, while mini-MAVS acts to inhibit the activation of full-length MAVS by preventing MAVS aggregation on the mitochondrial membrane (37). Immunoblot assay showed that the protein levels of full-length MAVS decreased in cells infected with rSA11, RRV, and OSU strain, but was largely unchanged in Wa-infected cells (Fig. 1).

**Fig 2:**
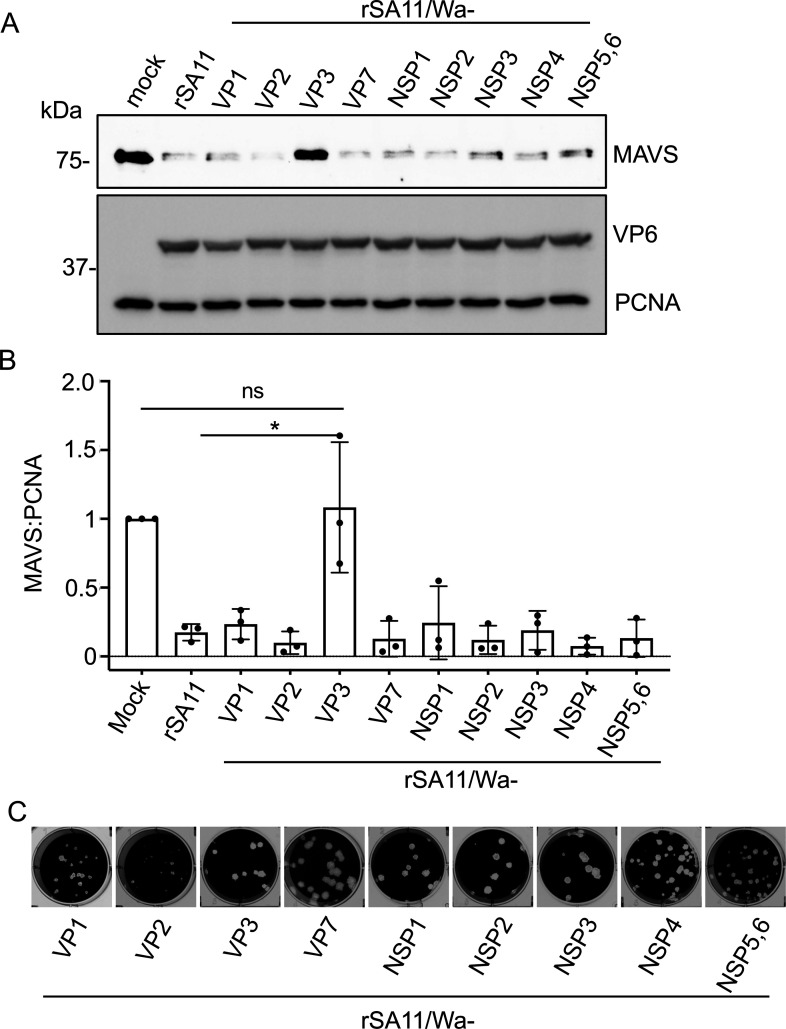
Level of endogenous MAVS in cells infected with rSA11/Wa monoreassortant viruses. (**A**) HT-29 cells were mock infected or infected with wild-type rSA11 virus (rSA11) or a rSA11/Wa reassortant virus (MOI = 5). Immunoblot assay was used to detect MAVS, VP6, and PCNA in cell lysates prepared at 8 h p.i. Results are representative of three independent experiments. (**B**) Quantification of endogenous MAVS levels from three independent experiments. Band intensities of MAVS were normalized to the PCNA loading control. The ratio of MAVS to PCNA for mock-infected cells was set to 1. *P* value was determined by unpaired Student’s *t*-test. ns, not significant; *, *P* ≤ 0.05. (**C**) Plaques of rSA11/Wa reassortant viruses formed on MA104 cell monolayers (4 d p.i.).

To understand the basis for the difference in the abilities of the virus strains to induce MAVS degradation, nine rSA11/Wa monoreassortant viruses, each carrying a Wa genome segment in the genetic background of rSA11, were generated by reverse genetics. Despite numerous attempts, it was not possible to recover the monoreassortant rSA11/WaVP4 and rSA11/WaVP6 viruses. Analysis of the nine rSA11/Wa monoreassortant viruses showed that rSA11/WaVP3 was the only virus not able to induce detectable MAVS degradation in the infected cells (Fig. 2A and B). This virus expressed a comparable level of VP6 protein to that of wild-type rSA11 virus and exhibited similar plaque sizes to other monoreassortants (Fig. 2C), suggesting its defect in inducing MAVS degradation did not result from compromised viral fitness. Together, these results suggest that VP3 plays a role in MAVS degradation and the VP3 protein of some, but not all, rotavirus strains can induce MAVS degradation.

### NSP1 is not required for SA11 rotavirus-induced MAVS degradation

**Fig 3:**
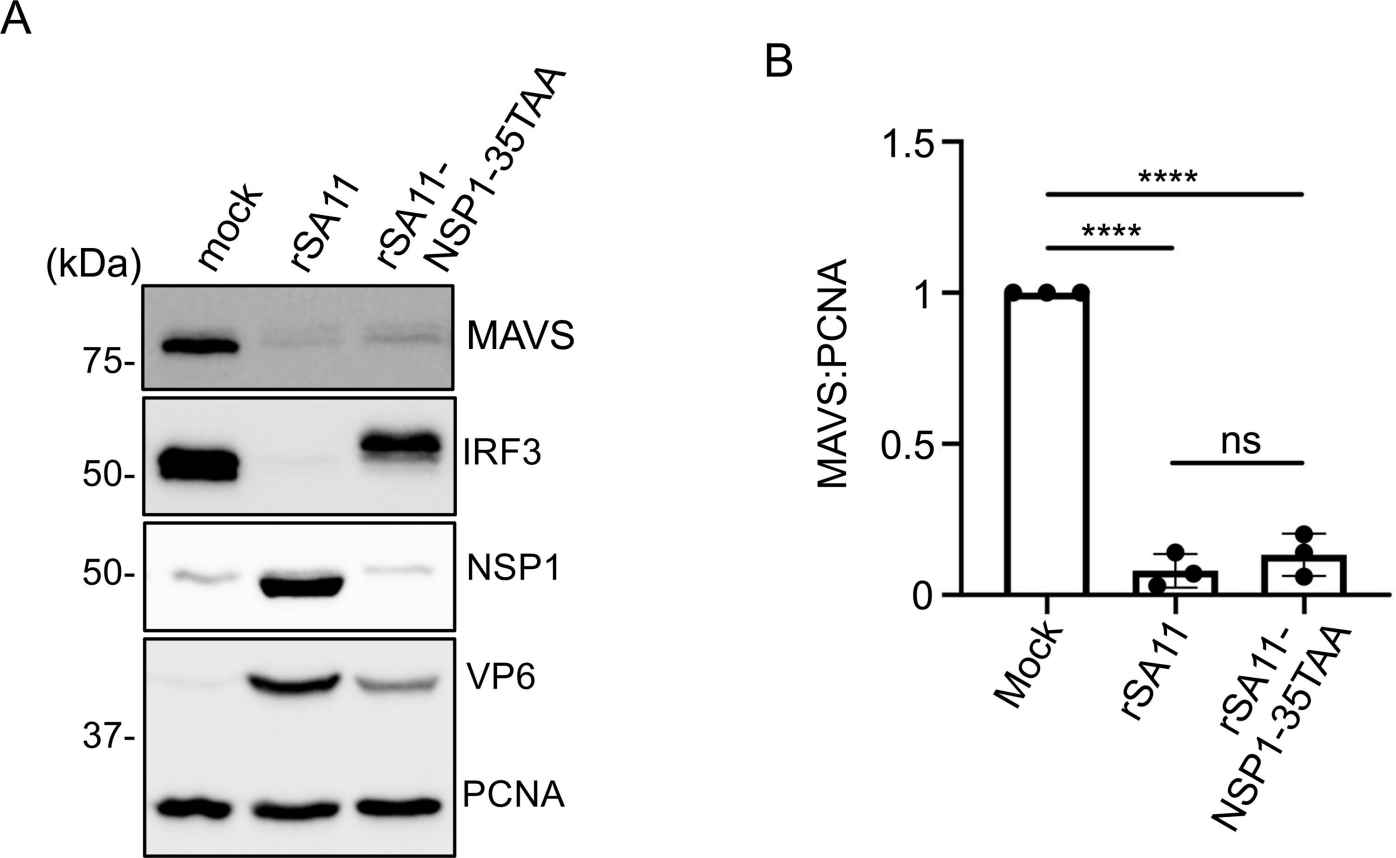
Level of endogenous MAVS in HT-29 cells infected with wild-type or NSP1-defective rSA11 virus. (**A**) HT-29 cells were mock infected or infected with the wild-type rSA11 virus (rSA11) or a mutant rSA11 virus encoding only the first 35 amino acids of NSP1 (rSA11-NSP1-35TAA) at an MOI of 5. Immunoblot assay was used to detect MAVS, IRF3, NSP1, VP6, and PCNA in cell lysates prepared at 10 h p.i. Results are representative of three independent experiments. (**B**) Levels of MAVS were quantified with a Bio-Rad ChemiDoc imaging system. Band intensities for MAVS were normalized to the PCNA loading control. The ratio of MAVS to PCNA for mock-infected cells was set to 1. *P* values were determined using the unpaired Student’s *t*-test. ****, *P* ≤ 0.0001; ns, not significant.

NSP1 has been shown to induce the degradation of multiple host proteins, including IRF3, β-TrCP, and RNase L, by hijacking the host ubiquitin-proteasome pathway (24, 38–40). Previous reports indicated that overexpression of NSP1 of the OSU, Wa, Ku, and DS-1 virus strains caused a decrease in MAVS levels in 293T cells (29). By contrast, another report indicated that transient expression of a GFP-tagged NSP1 of the RRV strain had minimal impact on MAVS levels in MA104 cells (30). To test whether NSP1 is required for rotavirus-induced MAVS degradation, a mutant rSA11 virus expressing only the first 35 residues of the 496 amino-acid NSP1 protein (rSA11-NSP1-35TAA) was generated by reverse genetics and examined for the ability to induce MAVS degradation (24). Although this virus grew less efficiently (24) and expressed lower levels of VP6 protein, the virus was able to reduce MAVS levels, indicating that NSP1 is not required for rSA11-induced MAVS degradation in HT-29 cells (Fig. 3A and B).

A shift up was noted in the electrophoretic migration pattern of IRF3 present in rSA11-NSP1-35TAA-infected cells, as compared to mock-infected cells. The shift up likely results from the phosphorylation of IRF3 in rSA11-NSP1-35TAA-infected cells, since this virus expresses a truncated form of NSP1 that is not able to induce IRF3 degradation. Interestingly, IRF3 phosphorylation took place in rSA11-NSP1-35TAA-infected cells, despite the depletion of MAVS, a protein with a critical role in the canonical IFN signaling pathway. This finding is consistent with previous reports suggesting that IRF3 activation and IFN expression may be mediated through MAVS-independent processes (e.g., Toll-like receptor/TRIF pathway) in rotavirus cells (41).

### Proteasome inhibitor MG-132 restores MAVS levels in rotavirus-infected cells

**Fig 4:**
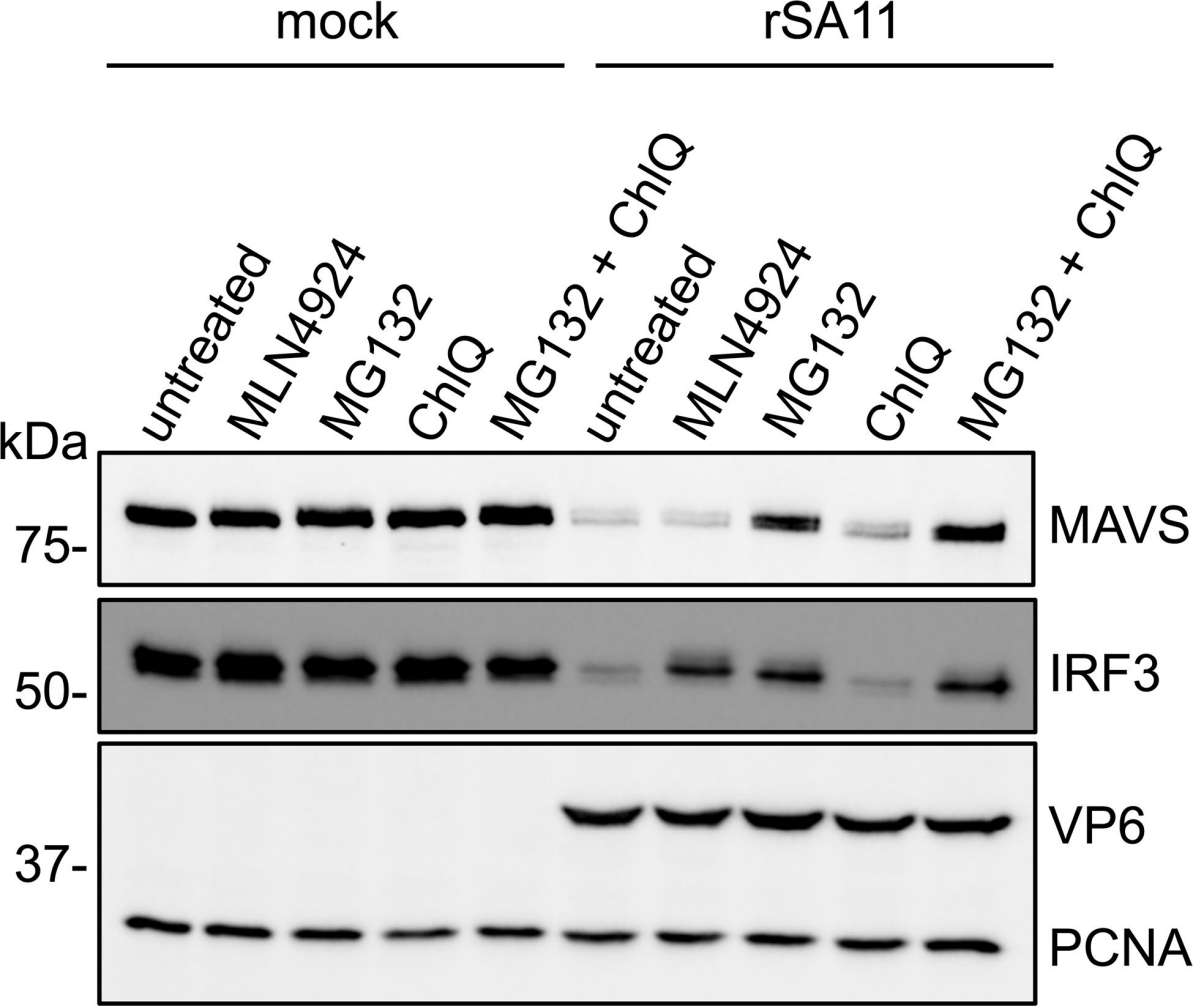
Level of endogenous MAVS in HT-29 cells treated with different inhibitors and infected with rSA11 virus. HT-29 cells were treated with the indicated inhibitors and mock infected or infected with wild-type rSA11 virus (MOI = 5). Cell lysates were prepared at 8 h p.i. and analyzed by immunoblot assay for MAVS, IRF3, VP6, and PCNA. UT, untreated; ChlQ, chloroquine. Blots are representative of two independent experiments.

Mixed evidence exists about the mechanism underlying rotavirus-induced MAVS degradation. One study proposed that NSP1 interacts with the CARD or TM domain of MAVS, causing ubiquitin-dependent proteasomal degradation of MAVS (29). Another study suggested that VP3 induced phosphorylation of a SPLTSS motif in the proline-rich region of MAVS (30). The phosphorylated motif was presumed to be recognized by host E3 ubiquitin ligase, resulting in K48-linked ubiquitination and proteasomal degradation of MAVS (30). To gain further insight into the basis of rotavirus-induced MAVS depletion, we tested different inhibitors for the ability to rescue MAVS levels in rotavirus-infected HT-29 cells. This analysis showed that the proteasome inhibitor MG-132, but not the Cullin-RING E3 ligase (CRL) inhibitor MLN4924 nor the lysosome inhibitor chloroquine, prevented the degradation of MAVS in cells infected with rSA11 virus, suggesting MAVS is degraded by the proteasome (Fig. 4). MLN4924 inhibits CRLs, the largest family of E3 ubiquitin ligases, by blocking neddylation, a post-translational modification required for CRL activation (42). In view of the fact that MLN4924 did not abolish rotavirus-induced MAVS degradation, the involvement of other E3 ubiquitin ligases in the attack on MAVS needs to be considered.

### Intracellular localization of VP3 in rotavirus-infected cells

**Fig 5:**
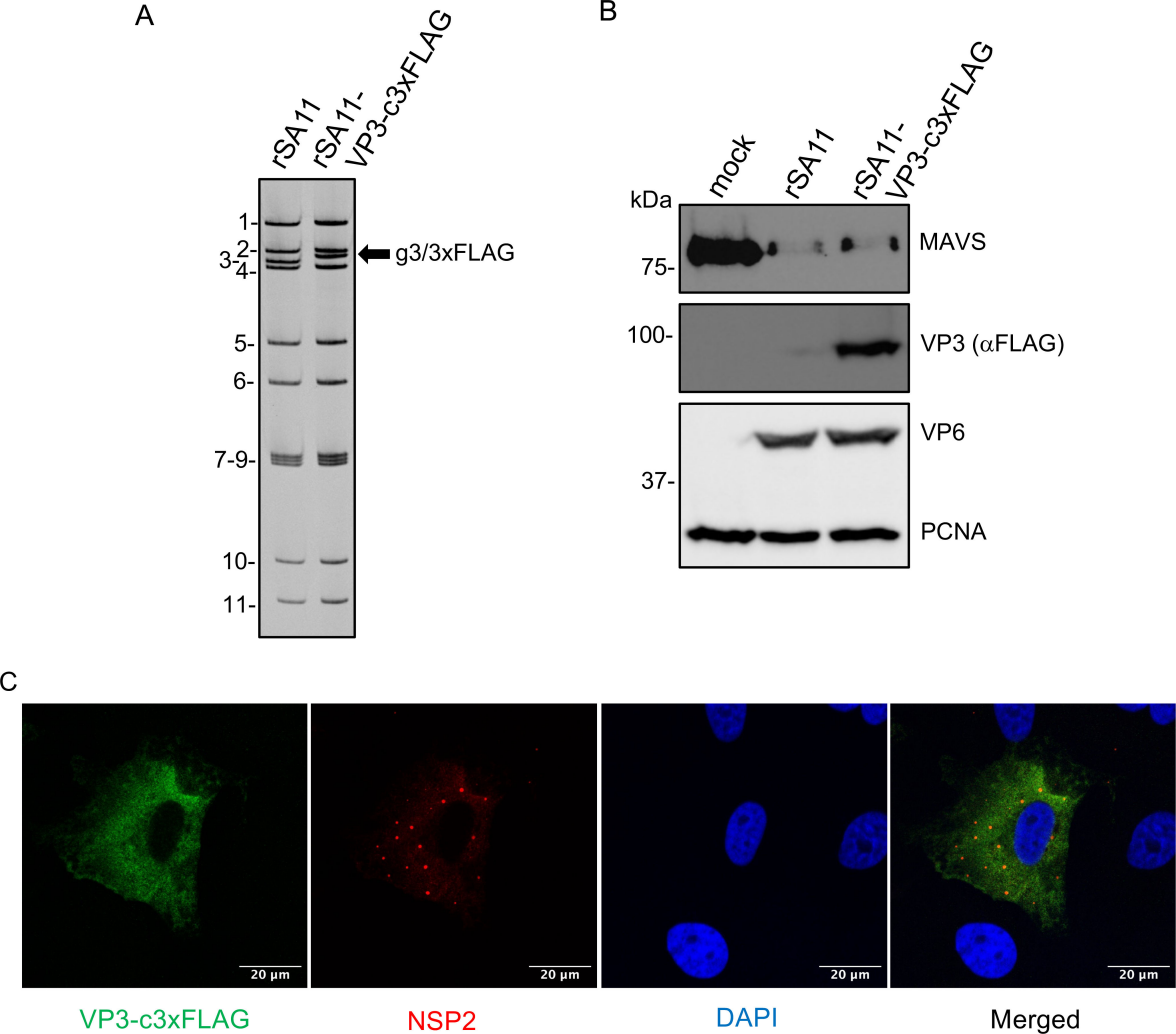
Intracellular localization of VP3. (**A**) Electrophoretic profile of the dsRNA genome segments of wild-type rSA11 virus and the mutant rSA11-VP3-c3xFLAG virus. Positions of rSA11 genome segments 1–11 are labeled. Also indicated is the position of the modified g3/3xFLAG genome segment (black arrow). (**B**) HT-29 cells were mock infected or infected with the indicated virus (MOI of 5). Immunoblot analysis of MAVS and 3xFLAG-tagged VP3 in mock- or virus-infected cell lysates prepared at 8 h p.i. (**C**) Immunofluorescence assay detecting 3xFLAG-tagged VP3 (anti-FLAG antibody, green), the NSP2 component of viroplasms (anti-NSP2 antibody, red), and nuclei (DAPI, blue) in MA104 cells infected with rSA11-VP3-3xFLAG virus and fixed at 7 h p.i. Scale bars represent 20 µm.

Little is known about the localization of VP3 in rotavirus-infected cells due to a lack of suitable VP3 antibodies to use in immunofluorescence assays. To overcome this issue, a recombinant rSA11 virus expressing VP3 with a C-terminal 3xFLAG tag was generated by reverse genetics. As shown by the RNA gel electrophoresis, the genome of this virus (rSA11-VP3-c3xFLAG) contained a 2.7 kb segment 3 dsRNA (encoding VP3) which migrated slightly slower than the 2.6 kb segment 3 of the wild-type rSA11 virus (Fig. 5A). Immunoblot analysis of the cell lysates prepared from rSA11-VP3-c3xFLAG-infected cells confirmed that the virus expresses a VP3 protein with a 3xFLAG tag. The reduced MAVS levels in cells infected with the rSA11-VP3-c3xFLAG virus indicated that the addition of a C-terminal 3xFLAG tag did not prevent VP3 from inducing MAVS degradation (Fig. 5B). As a structural protein, VP3 is expected to accumulate in viroplasms where progeny particles assembly occurs (43). However, as a multifunctional protein, VP3 is more than a structural protein, as it also induces the degradation of mitochondrial protein MAVS and cleaves the 2–5A signaling molecule of the OAS-RNase L pathway *via* its PDE domain (26, 30, 44). Therefore, VP3 accumulation may not be restricted to viroplasms. To gain insight into sites of VP3 accumulation, MA104 cells were infected with rSA11-VP3-c3xFLAG, and at 7 h p.i., the location of VP3 was determined by immunofluorescence assay using the anti-FLAG antibody. The results showed that VP3 accumulation was not limited to viroplasms, unlike the major viroplasm component NSP2. Instead, VP3 was distributed throughout the cytosol consistent with functions in degrading the cytosolic components MAVS and 2–5A (Fig. 5C).

### VP3-mediated MAVS degradation suppresses type I and type III interferon responses

**Fig 6:**
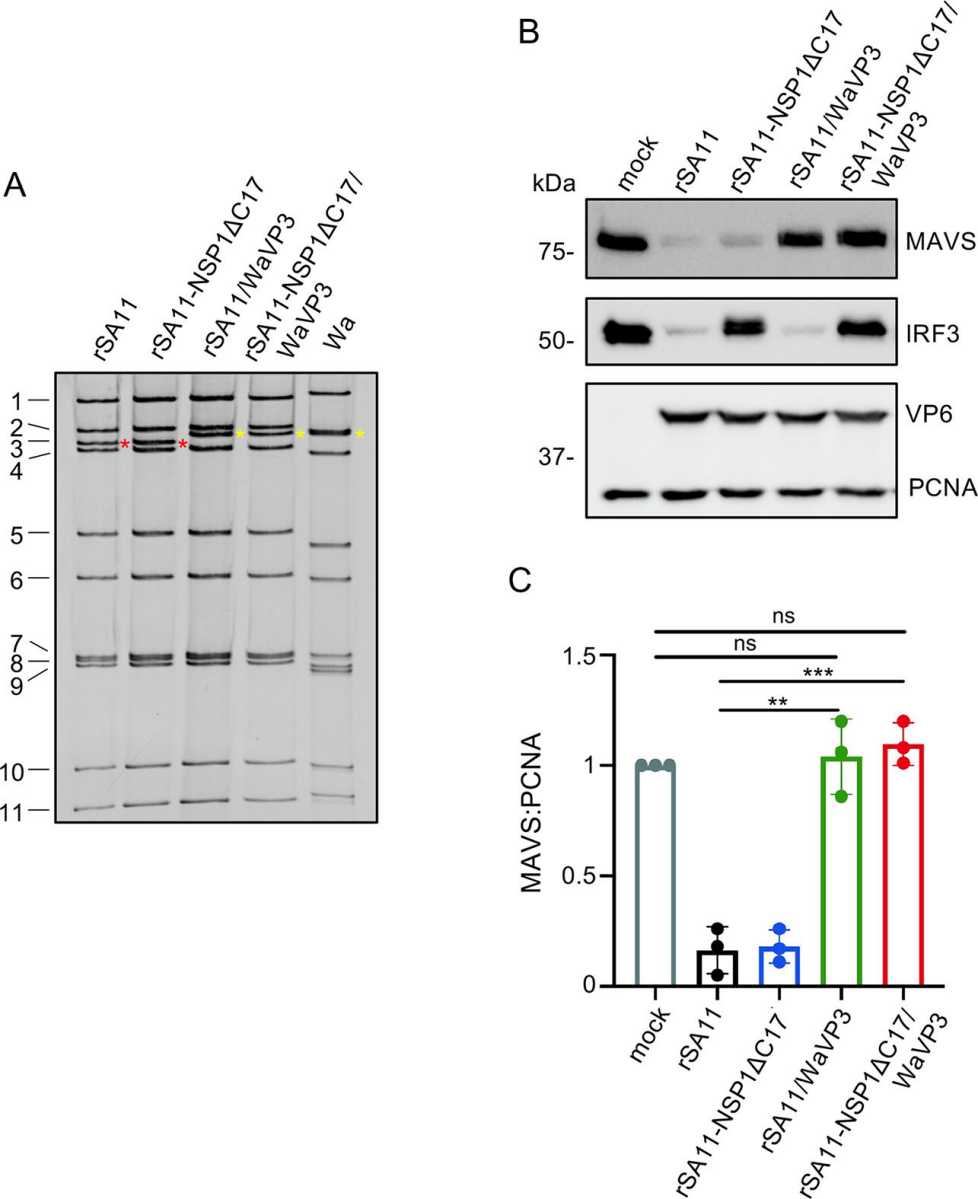
Contribution of rSA11 NSP1 and VP3 to the degradation of IRF3 and MAVS. (**A**) Electrophoretic profile of the dsRNA genomes of indicated viruses. Red and yellow asterisks note genome segment 3 of rSA11 and Wa strains, respectively. (**B**) HT-29 cells were mock infected or infected with the indicated rotavirus strain (MOI of 5). Immunoblot assay was used to detect MAVS, IRF3, VP6, and PCNA in cell lysates prepared at 8 h p.i. (**C**) Quantification of endogenous MAVS levels from three independent experiments. Band intensities of MAVS were normalized to the PCNA loading control. The ratio of MAVS to PCNA for mock-infected cells was set to 1. *P* values were determined by unpaired Student’s *t*-test. ns, not significant; **, *P* ≤ 0.01; ***, *P* ≤ 0.001.

**Fig 7:**
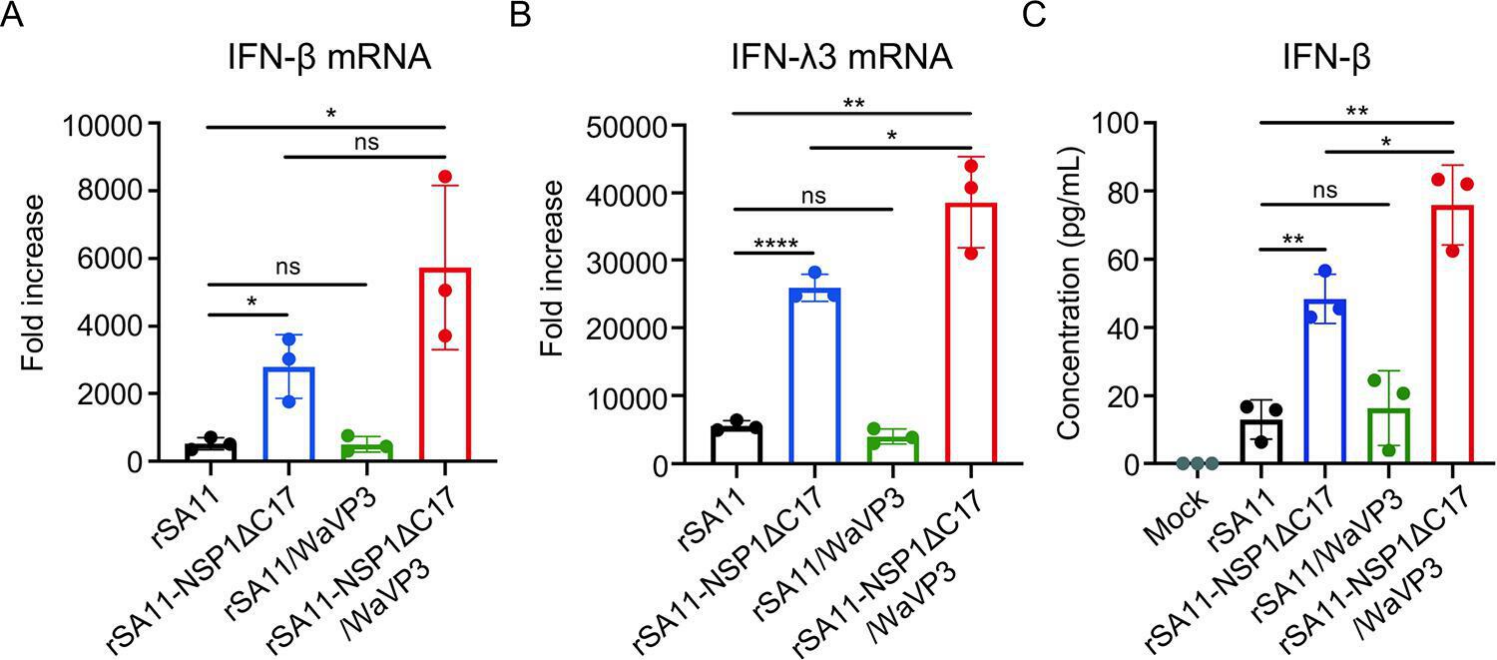
Increased IFN-β and IFN-λ3 expression in HT-29 cells infected with mutant rotaviruses failing to induce IRF3 and MAVS degradation. HT-29 cells were mock infected or infected with the indicated rotavirus (MOI = 5) and harvested at 8 h p.i. (**A, B**) RNA was extracted and reverse-transcribed using random primers. Fold increase in IFN-β and IFN-λ3 mRNA levels relative to GAPDH mRNA levels was quantified by normalizing to that of mock-infected cells. Figures are representative of three independent experiments. *P* values were determined by unpaired Student’s *t-*test. ns, not significant; *, *P* ≤ 0.05; **, *P* ≤ 0.01; ****, *P* ≤ 0.0001. (**C**) Culture media from HT-29 cells mock infected or infected with indicated viruses was recovered at 8 h p.i. and analyzed by ELISA for levels of IFN-β. The results were from three independent experiments. *P* values were determined by unpaired Student’s *t*-test. ns, not significant; *, *P* ≤ 0.05; **, *P* ≤ 0.01.

MAVS and IRF3 are pivotal proteins in the IFN signaling pathway (45). NSP1 of simian and several other animal strains has been shown to inhibit IFN production by inducing IRF3 degradation. But not all NSP1 proteins target IRF3. Instead, the NSP1 proteins of most human and porcine strains target β-TrCP for degradation rather than IRF3 (23, 40). Thus, we reasoned that VP3-mediated MAVS depletion may serve as an alternative strategy for suppressing IFN production for virus expressing NSP1 protein not able to degrade IRF3. To evaluate the contribution of NSP1 and VP3 to the inhibition of IFN production, we generated mutant rSA11 viruses that lacked the ability to degrade either IRF3 or MAVS or both. Previous studies demonstrated that a rSA11 virus expressing NSP1 with a C-terminal deletion of 17 residues (rSA11-NSP1ΔC17) is unable to induce IRF3 degradation (24). We also knew from the results presented above (Fig. 2A) that rSA11 virus expressing WaVP3 (rSA11/WaVP3) was less efficient in inducing MAVS degradation compared to other rotaviruses (Fig. 2A). To generate a virus lacking both IRF3- and MAVS- targeting activities, reverse genetics was used to produce a rSA11 virus expressing WaVP3 and SA11 NSP1ΔC17 (rSA11-NSP1ΔC17/WaVP3). RNA gel electrophoresis showed that the rSA11/WaVP3 and rSA11-NSP1ΔC17/WaVP3 viruses contained genome segment 3 of the Wa strain which migrated slower than that of the rSA11 strain (Fig. 6A). Immunoblot assay confirmed that viruses expressing NSP1ΔC17 lost the ability to degrade IRF3 and that viruses expressing WaVP3 did not induce MAVS degradation (Fig. 6B and C). Subsequently, the levels of IFN-β (type I IFN) and IFN-λ3 (type III IFN) mRNA were determined in HT-29 cells infected with these viruses using RT-qPCR. Consistent with previous studies (20), wild-type rSA11 suppressed the expression of both type I and type III IFN mRNA, whereas rSA11 virus producing NSP1ΔC17 activated IFN-β and IFN-λ3 expression. Notably, the virus unable to degrade either IRF3 or MAVS (rSA11-NSP1ΔC17/WaVP3) activated IFN expression at levels greater than rSA11-NSP1ΔC17, suggesting VP3-induced MAVS degradation may contribute significantly to rotavirus inhibition of IFN expression. However, we did not observe elevated IFN responses in cells infected with the rSA11/WaVP3 virus, although this virus was incapable of causing MAVS depletion (Fig. 7A and B). To test whether the increase in IFN-β transcript levels was correlated with increased levels of IFN-β protein expression, ELISA was used to quantify the concentration of IFN-β in the medium of infected HT-29 cells. Consistent with the RT-qPCR results, we detected higher levels of IFN-β in the medium of cells infected with rSA11-NSP1ΔC17 and rSA11-NSP1ΔC17/WaVP3 virus than in the medium of cells infected with wild-type rSA11 or rSA11/WaVP3 virus. The cells infected with the rSA11-NSP1ΔC17/WaVP3 virus released more IFN-β than those infected with the rSA11-NSP1ΔC17 virus (Fig. 7C).

### Temporal relationship between IRF3 and MAVS degradation during rotavirus infection

**Fig 8:**
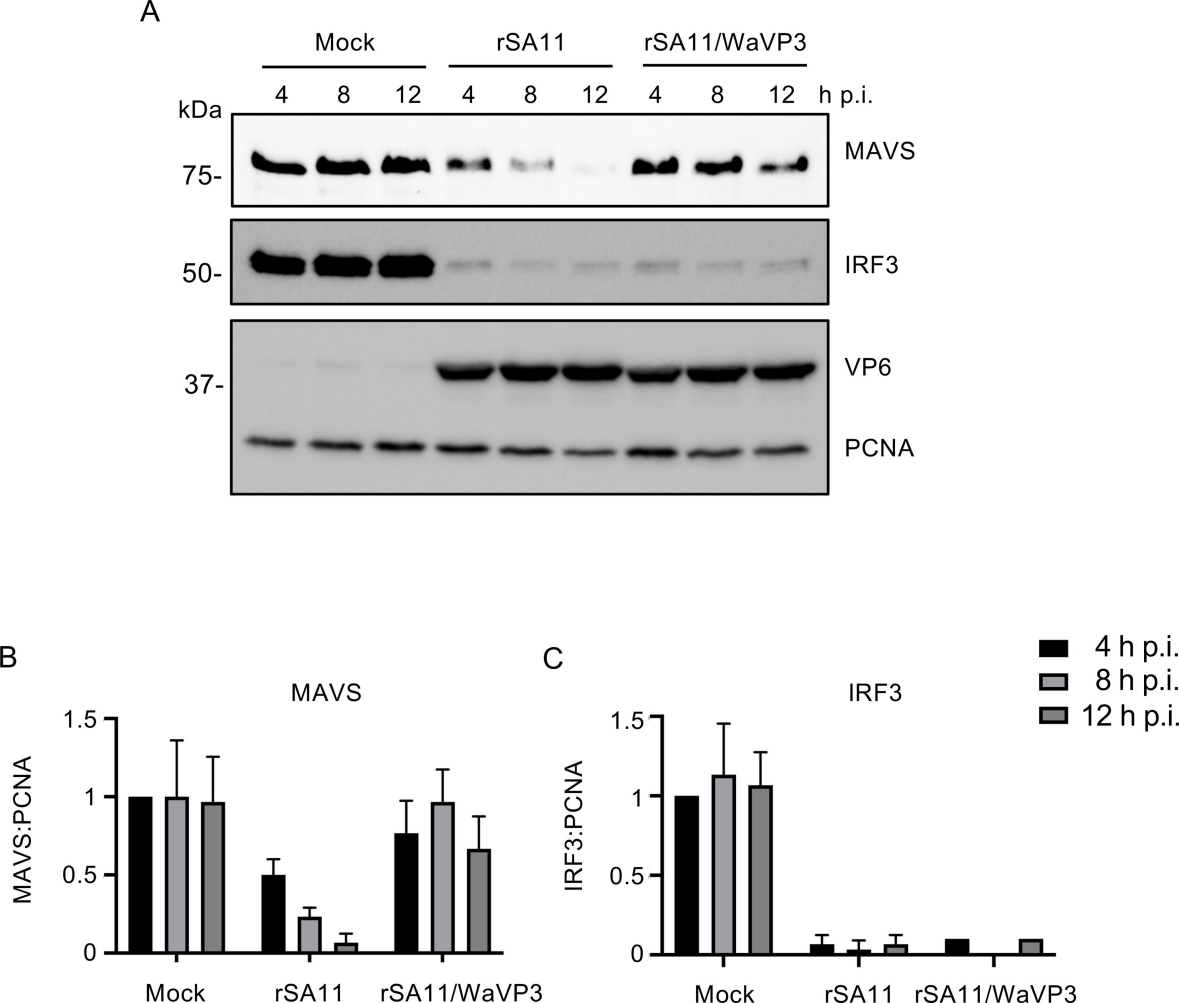
Time course of MAVS and IRF3 degradation in rotavirus-infected cells. (**A**) HT-29 cells were mock infected or infected with the indicated rotavirus (MOI = 5). Lysates prepared from cells at 4, 8, and 12 h p.i. were analyzed by immunoblot assay for MAVS, IRF3, VP6, and PCNA. (**B, C**) Levels of MAVS, IRF3, and PCNA were determined using ImageJ software. Band intensities of MAVS or IRF3 were normalized to the loading control PCNA. The ratio of MAVS or IRF3 to PCNA in mock-infected cells harvested at 4 h p.i. was set to 1. Results are from three independent experiments.

Although MAVS levels were relatively unchanged in cells infected with rSA11/WaVP3, this was not correlated with increased levels of IFN-β and IFN-λ3 mRNA (Fig. 7A and B). One possible explanation was that NSP1-induced degradation of IRF3 overshadowed the contribution of VP3-mediated MAVS degradation. To examine this possibility, the levels of MAVS and IRF3 presented in HT-29 cells infected with rSA11 and rSA11/WaVP3 were assessed during the course of infection. Immunoblot analysis showed that IRF3 degradation was nearly complete by 4 h p.i. in rSA11 and rSA11/WaVP3-infected cells (Fig. 8). By contrast, only ~50% of MAVS was degraded by 4 h p.i. in rSA11-infected cells. In the rSA11-infected cells, levels of MAVS degradation increased to 12 h p.i., when little MAVS remained (Fig. 8). These results suggest that NSP1-mediated IRF3 degradation is more efficient than VP3-induced MAVS degradation. For those rotavirus strains that are unable to subvert IRF3 activation, their VP3 may provide an alternative mechanism for dampening IFN production through MAVS degradation. Although rSA11/WaVP3 possesses a functional NSP1, it did not cause significant MAVS depletion even late in infection (12 h p.i.) (Fig. 8), confirming that NSP1 does not contribute to MAVS degradation. As MAVS was not completely degraded until 12 h p.i., the remaining MAVS present early in infection (4 and 8 h p.i.) might account for IRF3 phosphorylation in cells infected with NSP1 mutant virus (rSA11-NSP1-35TAA) (Fig. 3A).

### Increased type I and type III IFN levels did not affect viral replication in HT-29 cells

**Fig 9:**
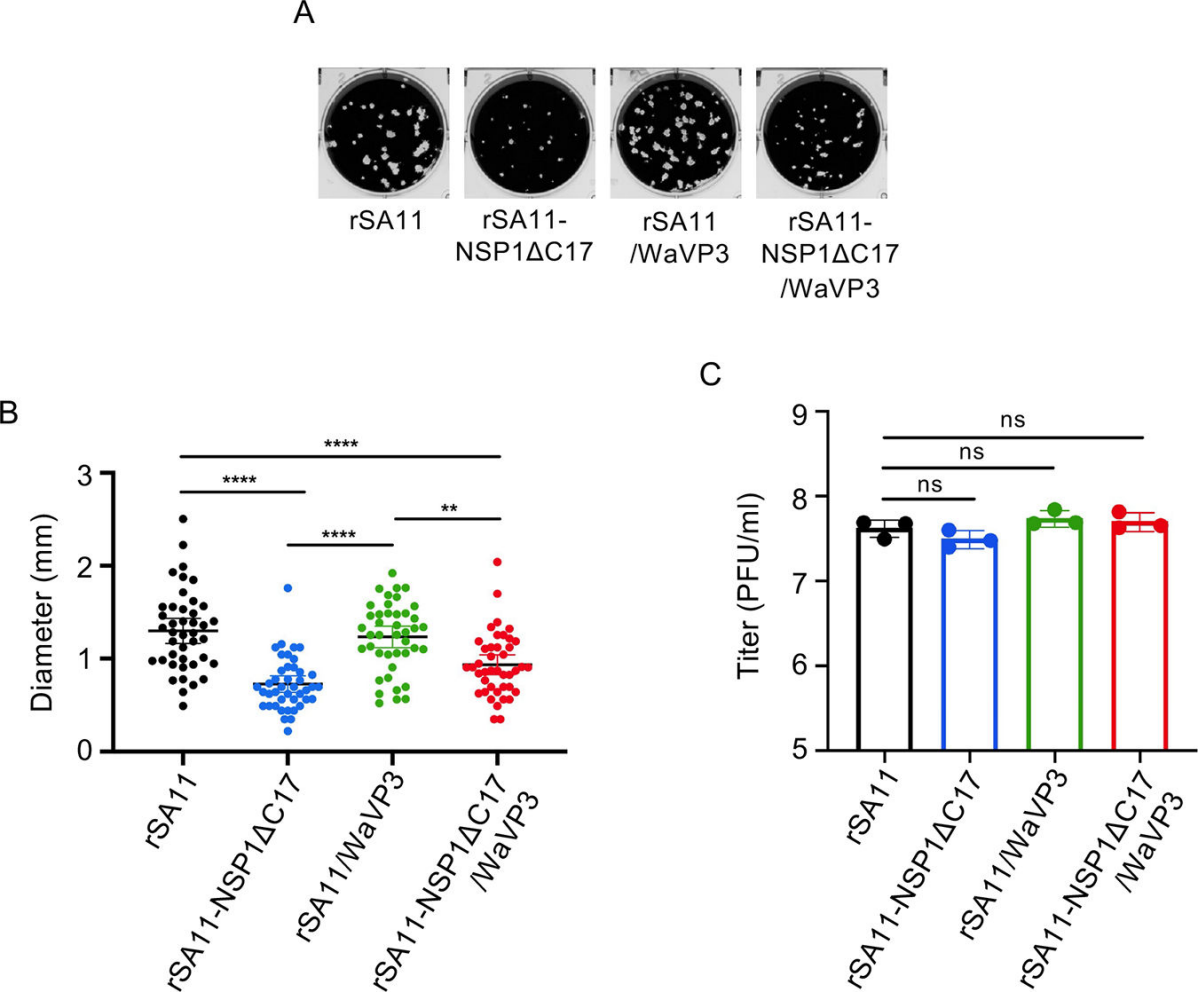
Growth properties of wild-type rSA11, rSA11-NSP1ΔC17, rSA11/WaVP3, and rSA11-NSP1ΔC17/WaVP3 viruses. (**A**) Plaques formed by wild-type and mutant rotaviruses on MA104 cells at 4 d p.i. (**B**) Diameter of plaques formed by wild-type and mutant rotaviruses. The diameters of 40 plaques were measured by ImageJ software from three independent experiments. Lines define the mean ± 95% confidence intervals. Significance values were determined using a one-way ANOVA test. **, *P* ≤ 0.01; ****, *P* ≤ 0.0001. (**C**) HT-29 cells were infected with indicated viruses at an MOI of 1 and harvested at 24 h p.i. Virus titers in cell lysates were determined by plaque assay on MA104 cells. The titer difference was analyzed using an unpaired Student’s *t*-test. ns, not significant.

Several studies suggest that IFN inhibits the spread of rotavirus in cell cultures and mice (20). To test whether increased IFN responses affected their fitness in cell culture, we compared the plaque sizes of rotaviruses able to induce IRF3 or MAVS degradation, or both, with those that cannot. The results showed that rSA11 and rSA11/WaVP3, both of which efficiently suppress IFN expression (Fig. 7), formed plaques with similar sizes (Fig. 9A and B). By contrast, rSA11-NSP1ΔC17 and rSA11-NSP1ΔC17/WaVP3, which fail to suppress IFN produced plaques with significantly smaller sizes (Fig. 9A and B). Although rSA11-NSP1ΔC17 and rSA11-NSP1ΔC17/WaVP3 exhibited reduced plaque sizes, they grew to titers comparable to wild-type rSA11 and rSA11/WaVP3 virus in HT-29 cells (Fig. 9C). Thus, the failure of rSA11-NSP1ΔC17 and rSA11-NSP1ΔC17/WaVP3 to suppress IFN expression may impede the ability of these viruses to undergo cell-to-cell spread without impacting virus replication within the infected cell.

## DISCUSSION

Innate immunity serves as a primary line of defense against a myriad of invading pathogens (46). This includes responses to rotavirus infection, which are dependent on activation of the IFN signaling pathway by the mucosal epithelium to restrict viral growth and pathogenesis and to promote an effective adaptive immune response. Based on studies with human enteroid cultures and mouse model systems, it is evident that multiple IFNs, including Type I and III, are likely to play pivotal roles in restricting rotavirus infection (47). To suppress the IFN signaling pathway, rotavirus strains have been identified as capable of inducing the degradation of two of its critical components: MAVS and IRF3 (29–31). In this study, we have used recombinant rotaviruses expressing modified VP3 and NSP1 to characterize the role of these proteins in disrupting the IFN signaling pathway by causing the degradation of MAVS and IRF3. We found that a monoreassortant rSA11 virus expressing VP3 of the human Wa strain (rSA11/WaVP3) failed to induce MAVS degradation in a manner characteristic of wild-type rSA11 virus. Although lacking the ability to induce measurable degradation of MAVS, rSA11/WaVP3 retained the ability to degrade IRF3, indicating that VP3 does not have a necessary role in IRF3 degradation. Notably, the rSA11/WaVP3 virus did not induce levels of IFN expression that were higher than that of the wild-type rSA11 virus. Thus, the viral protein most likely responsible for suppressing IFN levels in cells infected with wild-type rotavirus is NSP1 and not VP3.

To better understand the degradation processes of MAVS and IRF3, we analyzed the endogenous levels of these proteins in HT-29 cells infected with rSA11 and rSA11/WaVP3 viruses over the course of infection. Consistent with reports from previous studies (29), a notable change in MAVS levels was noted as a factor of time, with approximately 50% of MAVS depleted by 4 h p.i. and a near complete loss of MAVS detected by 12 h p.i. (Fig. 8). By contrast, nearly all IRF3 was lost from rSA11-infected cells by 4 h p.i. This finding suggests that NSP1-mediated IRF3 degradation is a more efficient process than VP3-mediated MAVS degradation, stressing the likely primary function of NSP1 in antagonizing IFN signaling. The delayed course of MAVS degradation may result from the competing demands placed on VP3 in the infected cell. As a viral structural protein, VP3 is recruited to viroplasms and incorporated into progeny particles, possibly lowering its effective concentration in the cytosol, particularly early in infection. Moreover, VP3, as a viral capping enzyme and as a PDE antagonist of the OAS-RNase L pathway, may be directed to substrates other than MAVS, again reducing its effective concentration at early times of infection.

Unlike wild-type virus, rSA11/WaVP3 did not induce significant MAVS degradation even at 12 h p.i. This virus, despite containing a wildtype genome segment 5 (which encodes NSP1) of the SA11 strain, did not cause MAVS depletion, suggesting that NSP1 is not responsible for the attack on MAVS. In cells infected with a mutant rSA11 expressing only a 35 amino acid remnant of the NSP1 open reading frame (rSA11-NSP1-35TAA), MAVS depletion was still observed despite the fact that the virus produced a nonfunctional NSP1. This result indicates that NSP1 is dispensable for SA11-mediated MAVS degradation. Sequence analysis of different rotavirus strains has shown that the NSP1 protein is the most varied in sequence among the 12 rotavirus-encoded proteins (40). The sequence variation allows some, but not all, NSP1 proteins to target select host proteins for degradation. We cannot rule out the possibility that the NSP1 proteins of certain rotavirus strains may induce MAVS degradation, although none were noted in this study.

Rotaviruses display a high degree of species specificity. In general, rotaviruses in their homologous host replicate more efficiently and are more virulent than in a heterologous host. The basis of rotavirus host range restriction (HRR) is multifactorial (48–51). However, the IFN response is considered to represent a major host determinant of HRR, one capable of suppressing the growth of rotavirus strains in their unnatural hosts. For instance, in neonatal mouse pups, IFN-mediated antiviral responses provide only modest growth restriction to homologous murine EC rotavirus strain but impose substantial suppressive growth restriction on the replication of the heterologous simian RRV strain (52). Several rotavirus genome segments, including segment 3 (which codes for VP3), have been implicated in host range restriction (48–51). In mouse embryonic fibroblasts (MEFs), the heterologous simian RRV strain but not the homologous murine ETD strain, is sensitive to IFN-β treatment. A monoreassortant RRV strain carrying genome segment 3 of murine EW strain displayed reduced sensitivity to exogenous IFN treatment, suggesting VP3 might confer resistance to IFN in a rotavirus strain- and host-species-specific manner (27). But whether VP3-mediated MAVS degradation provides the basis for the linkage of HRR with genome segment 3 is still unclear (29). In this study, we found that simian rSA11, simian RRV strain, and porcine OSU strain, but not human Wa, strain caused MAVS depletion in human HT-29 cells. These results indicate that VP3-mediated MAVS degradation may not be viral strain- nor host-species-specific.

Precise localization of endogenous VP3 in rotavirus-infected cells has not been possible because of the lack of a suitable high-affinity antibody that can specifically recognize VP3, a protein expressed at low levels in the infected cell. To overcome this limitation, we used reverse genetics to generate a recombinant rSA11 virus expressing a C-terminal 3xFLAG-tagged VP3. By immunofluorescence staining and confocal microscopy, we found that VP3 is not restricted to viroplasms in rotavirus-infected cells but is also distributed throughout the cytosol. This distribution pattern is consistent with the observation that VP3 has functions that likely take place outside the viroplasms, such as degradation of MAVS and hydrolysis of 2–5A.

Many questions remain unaddressed concerning the mechanism used by VP3 to degrade MAVS. For example, is the same E3 ligase that mediates MAVS degradation related to the E3 ligases involved in the degradation of RNase L and IRF3? And, although some rotaviruses, including Wa, do not induce MAVS degradation, do these strains nonetheless employ alternative mechanisms of preventing MAVS function? Based on studies of VP3, it is apparent that a major role of the protein is, like NSP1, to cripple the antiviral IFN signaling pathway and the activities of key ISG products. Mapping of its functional domains indicates that VP3 contains an N-terminal kinase-like domain, four intermediate domains with associated guanylyltransferase and methyltransferase activities that direct RNA capping, and a C-terminal PDE domain that degrades 2–5A (25). Transient expression experiments suggest that the N-terminal domain of VP3 is responsible for interaction with MAVS. Hopefully, the recovery of additional recombinant viruses with mutated VP3 proteins, including the N-terminal domain, by reverse genetics will allow us to identify residues that are critical to MAVS degradation.

## MATERIALS AND METHODS

### Cells and viruses

HT-29 cells were grown in McCoy’s 5A medium with L-glutamine and sodium bicarbonate (HyClone) supplemented with 10% heat-inactivated fetal bovine serum (FBS, Gibco) and 1× (100 units/mL each) penicillin and streptomycin solution (Quality Biological). MA104 cells were cultured in Dulbecco’s modified Eagle’s minimal (DMEM) essential medium with 4.5 g/L glucose, L-glutamine, and sodium pyruvate (DMEM, Corning) supplemented 10% FBS and 1 × penicillin-streptomycin. Baby hamster kidney cells expressing T7 RNA polymerase (BHK-T7) were kindly provided by Dr. Ulla Buchholz (Laboratory of Infectious Diseases, NIAID, NIH). BHK-T7 cells were cultured in Glasgow minimal essential medium with L-glutamine (GMEM, Lonza) supplemented with 5% FBS, 10% tryptose-phosphate broth (Gibco), 2 × nonessential amino acids solution (Gibco), and 1 × penicillin-streptomycin solution (53). Geneticin G418 (Gibco) was included in Glasgow MEM at a final concentration of 1 mg/mL with every other round of cell passage. Rotavirus strains RRV, OSU, and Wa were kindly provided by Dr. Yasutaka Hoshino (Laboratory of Infectious Diseases, NIAID, NIH). Prior to infection, viruses were activated by incubation with 10 µg/mL porcine trypsin (type IX-S, Sigma-Aldrich) for 45 min at 37°C (54), unless otherwise indicated. RRV, OSU, Wa, and rSA11 strains were propagated in MA104 cells maintained in serum-free DMEM containing 0.5 µg/mL porcine trypsin and titered by fluorescence focus assay (54).

### Plasmids

pT7 plasmids expressing rotavirus SA11 (+)RNAs (pT7/VP1SA11, pT7/VP2SA11, pT7/VP3SA11, pT7/VP4SA11, pT7/VP6SA11, pT7/VP7SA11, pT7/NSP1SA11, pT7/NSP2SA11, pT7/NSP3SA11, pT7/NSP4SA11, and pT7/NSP5SA11) were kindly provided by Takeshi Kobayashi (Osaka University) through the Addgene plasmid repository (https://www.addgene.org/Takeshi_Kobayashi/). Twist cloning vectors containing full-length sequences of the 11 (+)RNAs of Wa rotavirus were synthesized by Twist Bioscience. The Wa (+)RNAs were transferred from Twist cloning vectors into pT7 plasmids using an In-Fusion cloning kit (Takara). GenBank accession numbers of the Wa (+)RNA sequences are provided in Table 1. The pCMV-NP868R plasmid was constructed as described by Philip et al. (55), and the pT7/SA11VP3-GSG-3xFLAG-3dup, pT7/SA11-NSP1-35TAA, and pT7/SA11NSP1-ΔC17 plasmids were described by Dai et al. (24).

**TABLE 1:** Plasmids used in constructing recombinant SA11 rotaviruses

Recombinant virus	Modified genome segment	Plasmid used to modify genome segment	Accession no. for modified segment	Virus source
rSA11/WaVP1	VP1	pT7/WaVP1	OK872249.1	This study
rSA11/WaVP2	VP2	pT7/WaVP2	OK872250.1	This study
rSA11/WaVP3	VP3	pT7/WaVP3	OK872251.1	This study
rSA11/WaVP7	VP7	pT7/WaVP7	OK872257.1	This study
rSA11/WaNSP1	NSP1	pT7/WaNSP1	OK872253.1	(24)
rSA11/WaNSP2	NSP2	pT7/WaNSP2	OK872256.1	This study
rSA11/WaNSP3	NSP3	pT7/WaNSP3	OK872255.1	This study
rSA11/WaNSP4	NSP4	pT7/WaNSP4	OK872258.1	This study
rSA11/WaNSP5,6	NSP5,6	pT7/WaNSP5,6	OK872259.1	This study
rSA11-NSP1-35TAA	NSP1	pT7/SA11-NSP1-35TAA	ON710975.1	(24)
rSA11-NSP1ΔC17	NSP1	pT7/SA11NSP1ΔC17 (gift from Siyuan Ding)		(24)
rSA11-NSP1ΔC17/WaVP3	NSP1	pT7/SA11NSP1-ΔC17	OQ927961	This study
	VP3	pT7/WaVP3	OK872251.1	
rSA11-VP3-c3xFLAG	VP3	pT7/SA11VP3-GSG-3xFLAG3dup	ON711282.1	(24)

### Generation of recombinant viruses

Recombinant SA11 rotaviruses were generated by reverse genetics according to the method of Philip et al. (53). Briefly, BHK-T7 monolayers in 12-well plates were transfected with 11 pT7 plasmids, each expressing one of the SA11 (+)RNAs. Transfection mixtures contained 850 ng of each of the pT7 plasmids, except for the pT7/NSP2SA11 and pT7/NSP5SA11 plasmids, which were used at 2.55 µg each. Transfection mixtures also contained 850 ng of pCMV-NP868R plasmid expressing the NP868R capping enzyme of African swine fever virus (55) and 2 uL of TransIT-LT1 (Mirus) transfection reagent per μg of total plasmid. To generate rSA11/Wa reassortant viruses, or rSA11 expressing mutant forms of NSP1 or FLAG-tagged VP3, SA11 pT7 plasmids were replaced with the indicated plasmids listed in Table 1. After recovery by plaque isolation (54), recombinant viruses were propagated in MA104 cells.

To prepare stocks of semi-purified viruses that were free of contaminating cytokines (e.g., IFNs) and other debris, rotavirus-infected cell lysates were subjected to three rounds of freeze-thaw and clarified by centrifugation at 1,000× *g* for 15 min at 4°C. The clarified lysates were mixed with an equal volume of lipid solvent (Vertrel XF, TMC Industries), vortexed extensively, and the emulsion centrifuged at 1,000× *g* for 15 min at 4°C to resolve aqueous and organic fractions. Virus in the aqueous fraction was recovered by pelleting through a 1 mL cushion of 35% sucrose (wt/vol) in Tris-buffered saline (TBS), pH 7.4, at 100,000× *g* for 2.5 h at 4°C using a Beckman SW28 rotor. The virus pellet was resuspended in TBS and the concentration of infectious virus in resuspended (semi-purified) preparations was determined by plaque assay (54).

### RNA gel electrophoresis

Portions of semi-purified virus preparations were treated with 1,000 U of RNase T1 stock (1,000 U/uL, ThermoFisher) for 30 min at 37°C. Viral dsRNA was recovered by TRIzol extraction using a Direct-zol RNA Miniprep Kit (Zymo Research). Purified viral RNA was mixed with RNA loading buffer (0.125 M Tris-HCl, pH 6.8, 50% glycerol, and 0.01% bromophenol blue), resolved by electrophoresis on handcast 10% polyacrylamide gels using Tris-Glycine-SDS running buffer, and stained with ethidium bromide. The dsRNA genome segments were detected using a Bio-Rad ChemiDoc imaging system. Sequences of modified dsRNA genome segments in recombinant viruses were confirmed by Sanger sequencing (Eurofins).

### Treatment with inhibitors

HT-29 cells were treated with the proteasome inhibitor, MG-132 (Cell Signaling Technology), the neddylation inhibitor, MLN4924 (MilliporeSigma), and the lysosomal protease inhibitor, chloroquine (MilliporeSigma) as previously described (24). Briefly, HT-29 cells were washed twice with phosphate-buffered saline (PBS) prior to infection, then overlaid with FBS-free McCoy’s 5A medium containing 1 µM MLN4924 or 20 µM MG-132. After virus adsorption, cells were washed with FBS-free McCoy’s 5A medium and overlaid with the same medium containing 1 µM MLN4924 or 20 µM MG-132. FBS-free McCoy’s 5A medium containing 50 µM chloroquine was overlaid onto HT-29 cells at 3 h p.i.

### Immunoblot assay

To prepare cell lysates for immunoblot analysis, mock-infected and rotavirus-infected HT-29 monolayers in 24-well plates were washed with cold PBS and scraped into 100 µL IP lysis buffer [50 mM Tris-HCl, 150 mM NaCl, 1% Triton X-100, 1 × complete ethylenediaminetetraacetic acid-free protease and phosphatase inhibitor cocktail (Pierce)]. Lysates were incubated on ice for 20 min and clarified by centrifugation at 15,000× *g* for 30 min at 4°C. Clarified protein samples were mixed with SDS-PAGE loading buffer (MilliporeSigma) and denatured by heating to 95°C for 5 min. Denatured protein samples were resolved by electrophoresis on 10% polyacrylamide gels and transferred onto nitrocellulose membranes. Proteins on the blots were detected by incubation with the following primary antibodies: rabbit polyclonal anti-MAVS (CST 3993, 1:2,000), rabbit monoclonal anti-IRF3 (CST 11904S, 1:2,000), mouse monoclonal anti-FLAG M2 (Sigma-Aldrich F1804, 1:2000), guinea pig anti-VP6 (Lot 53963, 1:2000), or rabbit monoclonal anti-PCNA (CST 13110S, 1:2000). Primary antibodies were detected using horseradish peroxidase (HRP)-conjugated secondary antibodies at 1:10,000 dilutions, including goat anti-mouse IgG (KPL), goat anti-guinea pig IgG (Thermo Fisher, A18769), or goat anti-rabbit IgG (KPL). Signals were developed using Clarity Western ECL substrate (Bio-Rad, 170–5060) and detected using a Bio-Rad ChemiDoc MP imaging system.

### Immunofluorescence staining assay

MA104 cells were seeded on glass coverslips in 24-well plates and mock infected or infected with rotavirus at an MOI of 2. At 7 h p.i., the cells were fixed by incubation with 4% paraformaldehyde for 30 min at room temperature and permeabilized by incubating with 0.1% Triton-X 100 in PBS for 10 min. After washing twice with PBS, cells were blocked by incubating the coverslips in PBS containing 5% bovine serum albumin (BSA) overnight at 4°C. The cells were then incubated in PBS containing 3% BSA and the primary antibodies, rabbit polyclonal anti-FLAG (Bethyl 190–102A, 1:500), and mouse monoclonal anti-NSP2 (Lot 171, 1:500) for 1 h at room temperature. After washing three times with PBS, the cells were incubated with secondary antibodies: goat anti-rabbit Alex 488 and goat anti-mouse Alex 594. After three rounds of washing, the coverslips were mounted using a ProLong Gold antifade mounting reagent containing DAPI (Invitrogen). Images were captured using a Leica SP8 confocal microscope with a 63× oil-immersion objective at the Indiana University Light Microscopy Imaging Center (https://lmic.indiana.edu/index.html) and processed using Image J software (https://imagej.net/ij/index.html).

### RT-qPCR

HT-29 cell monolayers in 24-well plates were mock infected or infected with rotavirus at an MOI of 5. At 8 h p.i., the monolayers were washed twice with cold PBS and then 500 µL of TRIzol reagent (Invitrogen) was placed on the monolayers. After incubation at room temperature for 5 min, RNA was extracted from the cell lysates using a Direct-zol RNA Miniprep Kit (Zymo Research). Portions (1 ug) of the recovered RNA were reverse transcribed to cDNA using a High-Capacity cDNA Reverse Transcription Kit (Applied Biosystems) with random primers. The amount of cDNA product was quantified by qPCR using PowerUp SYBR Green Master Mix (Applied Biosystems) and gene-specific primers (Table 2). Fold changes in the levels of human IFN-β and IFN-λ3 mRNA relative to glyceraldehyde 3-phosphate dehydrogenase (GAPDH) mRNA were quantified by comparing them to that of mock-infected samples using the threshold cycle (ΔΔCt) method.

**TABLE 2:** Primers used for RT-qPCR gene expression analysis

	Forward	Reverse
IFN-β	ATGACCAACAAGTGTCTCCTCC	GGAATCCAAGCAAGTTGTAGCTC
IFN-λ3	TAAGAGGGCCAAAGATGCCTT	CTGGTCCAAGACATCCCCC
GAPDH	GGAGCGAGATCCCTCCAAAAT	GGCTGTTGTCATACTTCTCATGG

### Measurement of IFN-β levels by ELISA

HT-29 cell monolayers in 24-well plates were mock infected or infected with rotavirus at an MOI of 5. At 8 h p.i., media was collected from the cells analyzed for IFN-β levels by ELISA assay using a Human IFN-beta DuoSet ELISA kit (R&D Systems, DY814-05) according to the manufacturer’s instructions.

### Statistical analysis

For plaque size comparison, diameters of plaques were present as mean values and 95% confidence intervals. Significance values were calculated using a one-way analysis of variance with Bonferroni’s multiple-comparison test (GraphPad Prism 8). When comparing two groups of samples, the unpaired Student’s *t*-test was used (GraphPad Prism 8).
